# The superhealing MRL background improves muscular dystrophy

**DOI:** 10.1186/2044-5040-2-26

**Published:** 2012-12-05

**Authors:** Ahlke Heydemann, Kayleigh A Swaggart, Gene H Kim, Jenan Holley-Cuthrell, Michele Hadhazy, Elizabeth M McNally

**Affiliations:** 1 Department of Medicine, Section of Cardiology, 5841 S. Maryland, MC 6088, Chicago, IL, 60637, USA; 2 Department of Human Genetics, The University of Chicago, Chicago, IL, 60637, USA; 3 Current address: Department of Physiology and Biophysics, University of Illinois at Chicago, COMRB 2035, MC 901, 835 South Wolcott Ave, Chicago, IL, 60612-7352, USA

**Keywords:** Cardiomyopathy, Fibrosis, MRL, Muscular dystrophy

## Abstract

**Background:**

Mice from the MRL or “superhealing” strain have enhanced repair after acute injury to the skin, cornea, and heart. We now tested an admixture of the MRL genome and found that it altered the course of muscle pathology and cardiac function in a chronic disease model of skeletal and cardiac muscle. Mice lacking γ-sarcoglycan (*Sgcg*), a dystrophin-associated protein, develop muscular dystrophy and cardiomyopathy similar to their human counterparts with limb girdle muscular dystrophy. With disruption of the dystrophin complex, the muscle plasma membrane becomes leaky and muscles develop increased fibrosis.

**Methods:**

MRL/MpJ mice were bred with *Sgcg* mice, and cardiac function was measured. Muscles were assessed for fibrosis and membrane leak using measurements of hydroxyproline and Evans blue dye. Quantitative trait locus mapping was conducted using single nucleotide polymorphisms distinct between the two parental strains.

**Results:**

Introduction of the MRL genome reduced fibrosis but did not alter membrane leak in skeletal muscle of the *Sgcg* model. The MRL genome was also associated with improved cardiac function with reversal of depressed fractional shortening and the left ventricular ejection fraction. We conducted a genome-wide analysis of genetic modifiers and found that a region on chromosome 2 was associated with cardiac, diaphragm muscle and abdominal muscle fibrosis.

**Conclusions:**

These data are consistent with a model where the MRL genome acts in a dominant manner to suppress fibrosis in this chronic disease setting of heart and muscle disease.

## Background

Murphy Roths Large (MRL) mice are an inbred mouse strain noted to have enhanced healing ability. This MRL strain was initially discovered because of its rapid ability to heal ear holes [1,2]. The MRL strain’s capacity to rapidly recover from injury has been seen for both digit wounding and corneal scarring [3,4]. The MRL strain has been reported to reduce scar formation after acute cardiac injury, including freeze injury and coronary artery ligation [5,6]. However, other studies have suggested that larger scale acute cardiac injury cannot be overcome by the MRL strain’s healing capacity [7-11]. In those injury settings where the MRL background induces more rapid healing, multiple mechanisms have been implicated to explain this phenomenon including decreased scar formation, altered inflammatory response, reduced apoptosis, increased proliferation, improved remodeling and, in some settings, enhanced stem cell function [4,6,12-15]. Genetic data support that many different mechanisms account for enhanced healing since more than 40 different genetic loci have been associated with aspects of the healing phenotype [14,16].

The dystrophin complex is composed of membrane-associated proteins that mediate membrane stability in heart and skeletal muscle. Mutations that disrupt expression of dystrophin or its associated proteins the sarcoglycans proteins cause progressive cardiac and skeletal muscle degeneration in humans and mouse models. At the cellular level, the loss of dystrophin or the sarcoglycans leads to a disrupted sarcolemmal membrane that is abnormally leaky. Membrane permeability results in increased intracellular calcium that triggers proteolysis and necrosis. An inflammatory response also contributes to muscle degeneration [17]. Muscle contraction is thought to provoke submicroscopic disruption of the sarcolemma, and in the absence of a normal dystrophin complex, this leads to dysfunction and destruction of cardiomyocytes and skeletal myofibers. The reduction in contractile cells and the presence of fibrotic scar tissue within the heart led to reduced cardiac contractile function and congestive heart failure.

Mouse models of dystrophin or sarcoglycan mutations recapitulate the basic pathological defects seen in human forms of these genetic disorders. Mice lacking γ-sarcoglycan were engineered by removing the first coding exon of the *Sgcg* gene and model muscular dystrophy [18]. We previously introduced the *Sgcg* allele into the DBA/2J genetic background and found that this background confers a more severe phenotype [19]. The enhanced severity is seen as increased scar formation, measured as hydroxyproline (HOP) content because this modified amino acid is a marker of collagen. In skeletal muscle, increased Evans blue dye uptake is monitored to reflect membrane leakiness [20]. Using these assays, we showed that the DBA/2J genetic background worsened the disease process [19].

Because of its role in wound healing and the basic similarities between common forms of cellular injury and what is seen in muscular dystrophy and cardiomyopathy, we introduced the MRL genome into the *Sgcg* model. We hypothesized that the indolent pace of cellular damage in this disorder could be abated by the MRL background. *Sgcg* mice were bred to MRL mice to generate *Sgcg* mice on a mixed genetic background with 50% contribution of the MRL strain. This breeding strategy produced *Sgcg* mice with 50% genetic background from the MRL strain and 50% background of the DBA/2J strain, and these mice are referred to *Sgcg*^*MRL/D2*^. We found that a 50% contribution of the MRL genome reduced fibrosis in the heart and skeletal muscle, consistent with dominant genetic loci in the MRL background. Interestingly, the MRL genome did not consistently reduce membrane leak, suggesting that the MRL background does not exert its effect on myocyte membrane stability and instead acts downstream on remodeling. *Sgcg*^*MRL/D2*^ mice had improved cardiac function so that they were now similar to wild-type mice. We conducted a genome-wide scan using informative polymorphisms and identified a region on chromosome 2 that was associated with fibrosis in cardiac, diaphragm and abdominal muscles. These data demonstrate that genes within the MRL background are modifiers for cardiopulmonary involvement in muscular dystrophy.

## Methods

### Animals

The *Sgcg* mouse was previously bred for ten generations into the DBA/2J strain (000671, Jackson Laboratory, Bar Harbor, ME) [18,19]. MRL/MpJ (000486, Jackson Laboratory, Bar Harbor, ME) mice were bred to the *Sgcg* animals to generate *Sgcg*^+/−^ F1 mice on a 50% MRL/50% DBA/2J background. F1 mice were interbred to generate an F2 cohort. To increase the number of mutant animals, *Sgcg* mice from the F2 generation were bred again to produce additional *Sgcg* mice from an F3 generation. All mice were housed in uniform conditions in a single pathogen-free barrier facility. All animals used in this study were housed and treated in accordance with the standards set by the University of Chicago Animal Care and Use Committee. The number of mice used for histopathological analysis is shown in Table 1.

**Table 1:** Number of mice analyzed

**Trait**	***Genotypes***		
**Fibrosis**	***Sgcg***^***D2***^	***Sgcg***^***MRL/D2***^	***Sgcg***^***MRL/D2***^***, *32wk**
Quadriceps	25	124	18
Diaphragm	25	45	10
Heart	27	125	9
**Membrane leak**	***Sgcg***^***D2***^	***Sgcg***^***MRL/D2***^	***Sgcg***^***MRL/D2***^***, *32wk**
Quadriceps	37	149	15
Gluteus	11	76	15
Triceps	14	110	15
Abdominals	14	74	15
Gastrocnemius/soleus	14	111	15
**Central nuclei**	***Sgcg***^***D2***^	***Sgcg***^***MRL/D2***^	
Quadriceps	6	7	
Diaphragm	6	4	
**Fiber size variability**	***Sgcg***^***D2***^	***Sgcg***^***MRL/D2***^	
Quadriceps	7	9	
Diaphragm	6	4	

Mice were sacrificed at either 8 or 32 weeks for analysis. Muscles were used for either the Evans blue dye assay or the HOP assay. Dye uptake assays were performed on triceps muscles, gastrocnemius/soleus muscles and gluteus/hamstrings groups of muscles. Dye uptake was performed on half of each quadriceps muscle and half of the abdominal muscles since these muscles are of sufficient size to perform both dye uptake and fibrosis assays. Hydroxyproline (HOP) assays were performed on the diaphragm muscle, the cardiac ventricles isolated as a single unit, half of the abdominal muscles and half of each quadriceps muscle. The amount of muscle assayed varied with the size of the muscle and ranged from 30 mg for the diaphragm muscle to 300 mg for the gluteus/hamstring muscle group that included the semimembranosus, semitendinosus and biceps femoris.

### Evans blue dye uptake assay for membrane leak

Evans blue dye (Sigma, E-2129) was performed as described [19,21]. Evans blue dye (Sigma, E-2129) was dissolved in phosphate-buffered saline at 10 mg/ml and injected intraperitoneally at 5 μl/g body weight. Twenty to 40 h later, the tissues were harvested, finely minced, weighed and incubated at 55°C in 1 ml formamide for 2 h before spectrophotometric absorbance was measured at 620 nm [22,23]. Results are reported as absorbance/mg tissue.

### Hydroxyproline assay for fibrosis

The hydroxyproline (HOP) assay was performed as described [19,21,24]. The tissue was minced, weighed and hydrolyzed overnight in 2 ml of 6 M hydrochloric acid at 110°C. Ten μl of this hydrolysate was mixed with 150 μl isopropanol, then 75 μl of 1.4% chloramine-T (Sigma, St Louis, MO) in citrate buffer and oxidized at room temperature for 10 min. One ml of a 3:13 solution of Ehrlich’s reagent (3 g of 4-(dimethylamino) benzaldehyde, Sigma, St Louis, MO; 10 ml ethanol; 675 μl sulfuric acid) to isopropanol was added, mixed and incubated for 30 min at 55°C followed by extinction measurement at 558 nm. A standard curve (0–5000 nM, trans-4-hydroxy-L-proline, Sigma, St Louis, MO) was included in each assay. Results are reported as nM HOP/mg tissue.

### Immunofluorescence microscopy

Tissues were flash frozen in liquid nitrogen-cooled isopentane and stored at 80°C; 7-μm sections were cut on a cryostat and fixed to slides in ice-cold 100% methanol. The following antibodies were used: dystrophin NCL-DYS2 (Novocastra/Leica), PH3 04–817 (Millipore), CD3 MON1003-1 (Monsanto), caspase 3 (BD Biosciences), MAC1 BD557395 (BD Biosciences) and eMHC F1.652 (ATCC). The TUNEL kit was from Millipore. Central nuclei and fiber size variability was determined blinded to genotype by analyzing ten randomly chosen fields of 40× magnification. Fiber size variability was compared using each animal’s coefficient of variability (standard deviation/mean).

### Echocardiography

Twelve-week animals were evaluated by echocardiography as described [25,26]. Investigators were blinded to genotype. To avoid the stress associated with conscious restraint, anesthetized animals were studied. Anesthesia was induced by 1% isoflurane in a closed chamber (Ohmeda Fluotec 3; Matrix Medical, Orchard Park, NY) in 20% O_2_ delivered through a nose cone. Chest hairs were removed with a topical depilatory agent. Limb leads were attached for electrocardiogram gating, and the animals were imaged in the left lateral decubitus position with a Visual Sonics Vevo 770 machine using a 30-MHz high-frequency transducer. Body temperature was maintained using a heated imaging platform and warming lamps. Anesthesia was variably delivered to maintain heart rates throughout the procedure at a constant 380–420 beats per minute. Two-dimensional images were recorded in parasternal long- and short-axis projections, with guided M-mode recordings at the midventricular level in both views. LV cavity size and wall thickness were measured in at least three beats from each projection and averaged. LV wall thickness, interventricular septum (IVS) and posterior wall (PW) thickness, and internal dimensions at diastole and systole (LVIDd and LVIDs, respectively) were measured. LV fractional shortening [(LVIDd – LVIDs)/LVIDd] and relative wall thickness [(IVS thickness + PW thickness)/LVIDd] were calculated from the M-mode measurements.

### Genetic and statistical analysis

Single nucleotide polymorphisms (SNPs, *n* = 1,701) informative between the parental DBA/2J and MRL/MpJ strains were genotyped in 80 *Sgcg*^*MRL/D2*^F2-F4 *Sgcg* animals on the Illumina GoldenGate platform using the Mutation Mapping and Developmental Analysis Panel (MMDAP) [27] and the Mouse Universal Genotyping Array (GeneSeek, Neogen Corp., Lansing, MI) [28]. R package QTLRel was used to perform whole-genome quantitative trait locus (QTL) mapping for each of the membrane permeability and fibrosis phenotypes and using sex as a covariate [29,30]. Significance thresholds were determined by 1,000 permutation tests. The 1.5-LOD drop support interval was calculated using QTLRel. Tests of normality and other statistics were calculated in Prism (GraphPad). Build 37.1 was used for genomic analysis.

For HOP and dye uptake assays, data were analyzed by one-way, unpaired ANOVA with parametric methods followed by the Tukey multiple comparison post-test (Prism, Graphpad), and *p* < 0.05 was considered significant. Data from 197 *Sgcg* animals from an F3 intercross between the DBA/2J and 129T2/SvEmsJ backgrounds (*Sgcg*^*D2/129*^ ) was compared to the *Sgcg*^*MRL/D2*^cohort.

## Results

### A 50% contribution of the MRL background reduces fibrosis in Sgcg mice

**Figure 1:**
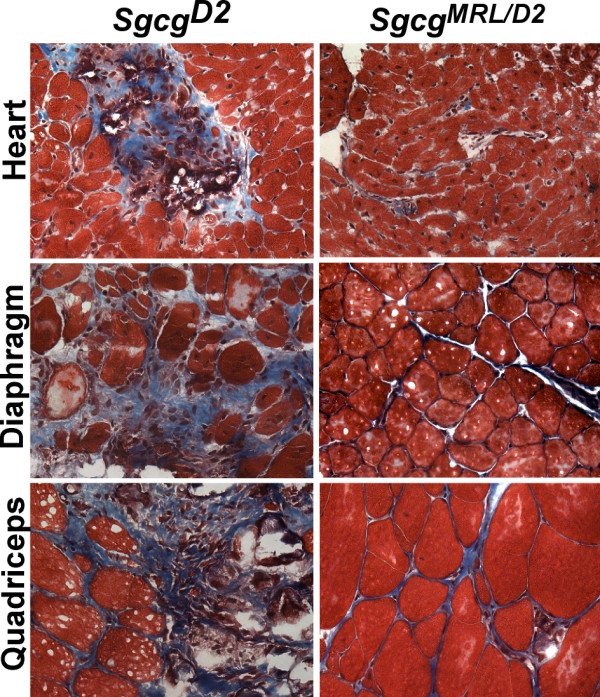
**The MRL genome suppresses fibrosis in the heart and muscles of *Sgcg***^***D2***^**mice.***Sgcg* mice lack the dystrophin-associated protein, γ-sarcoglycan, and when in the DBA/2J (D2) background have a more severe phenotype with enhanced membrane leak and fibrosis [21]. A 50% contribution of the MRL genome, referred to as *Sgcg*^*MRL/D2*^, suppressed fibrosis in both heart and skeletal muscle.

To assess the MRL contribution to muscular dystrophy, we used the MRL/MpJ substrain since it contains a wild-type *fas* allele [1]. We also used mice lacking γ-sarcoglycan (*Sgcg* null) since these mice are a model of limb girdle muscular dystrophy 2C [18] and on the DBA/2J background have a more severe phenotype, reminiscent of what is seen in humans [19,21]. MRL/MpJ mice were bred to *Sgcg* null and then interbred to generate *Sgcg* null mice with a 50% MRL/MpJ and 50% DBA/2J contribution. In *Sgcg* null mice, like all dystrophin complex-associated mutations, fibrosis and collagen deposition is increased in the heart and muscle, similar to what is seen in human patients with similar mutations [18,31]. A 50% contribution from the MRL background reduced fibrosis in *Sgcg* heart and muscles (Figure 1). In *Sgcg* hearts, fibrosis was often seen grossly as large patchy white areas and with the introduction of the MRL background visible fibrosis was diminished so that the hearts were indistinguishable from those of wild-type mice (Additional file 1: Figure S1). Of all the muscle groups analyzed, only diaphragm muscle retained any visible fibrosis. Diaphragm muscle is the most consistently damaged muscle in multiple mouse models, including this model, and the *mdx* model of Duchenne muscular dystrophy [32]. Therefore, it is possible that the degree of injury in this muscle overwhelms the healing properties of the MRL background.

**Figure 2:**
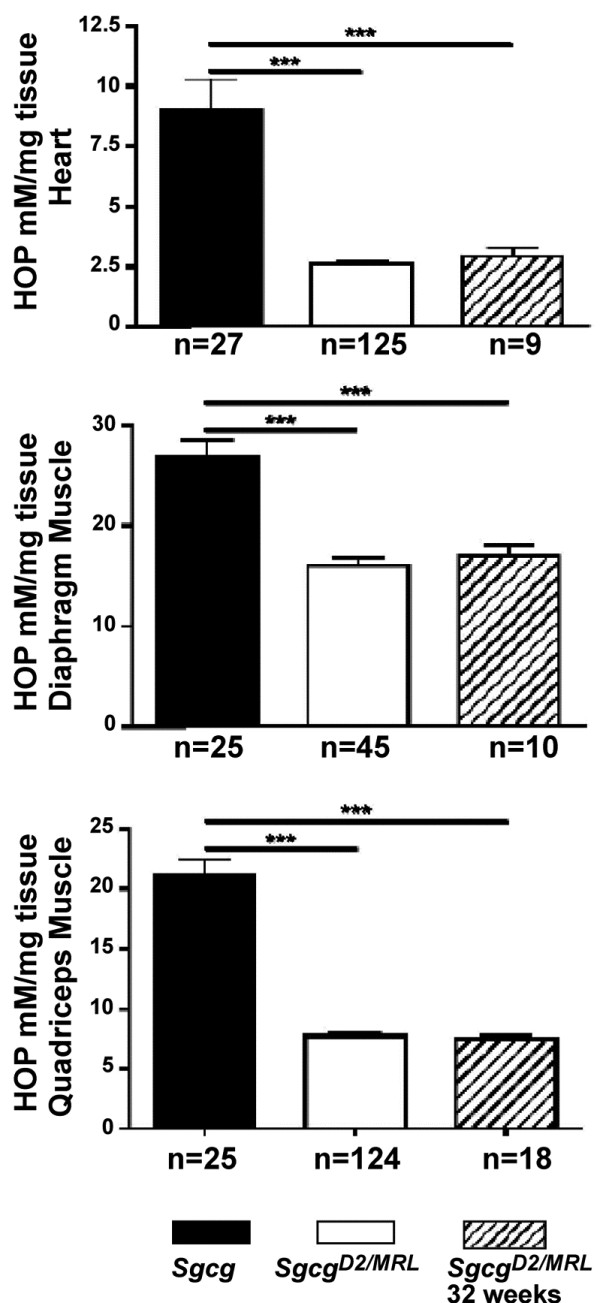
**The MRL genome quantitatively reduces fibrosis in *Sgcg *mice.** Hydroxyproline (HOP) is a measure of fibrosis and is represented on the y axis (mM/mg). In the heart, diaphragm and quadriceps muscles, fibrosis is significantly (*p* < 0.001) reduced in intercrossed *Sgcg*^MRL/D2^ (D2/MRL) animals compared to *Sgcg* in the D2 (D2) background at 8 weeks. Fibrosis remains significantly (***p < 0.001) reduced in *Sgcg*^*MRL/D2*^ animals at the 32-week time point.

**Figure 3:**
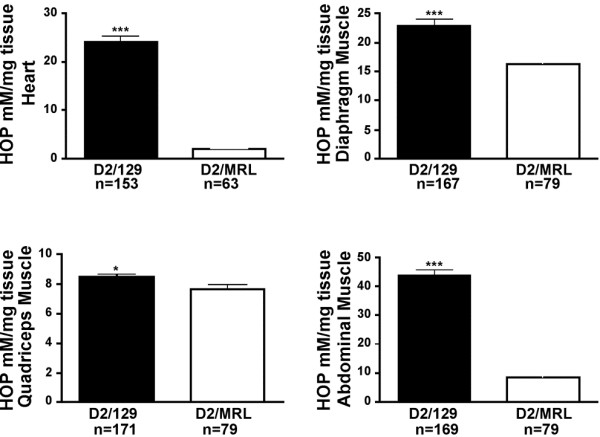
**The reduction of fibrosis in intercrossed *Sgcg *animals is specific to the MRL genome.** Hydroxyproline (HOP) is a measure of fibrosis and is represented on the y axis (mM/mg). Fibrosis is significantly reduced in the heart (***p < 0.001), diaphragm (*p* < 0.001), quadriceps (*p = 0.041) and abdominal muscles (*p* < 0.001) of *Sgcg* animals intercrossed in the D2/MRL background compared to those intercrossed in the D2/129 background.

We quantified fibrosis by measuring HOP content as an indicator of collagen in *Sgcg*^*D2*^ and *Sgcg*^*MRL/D2*^ muscles. *Sgcg*^*MRL/D2*^ mice have significantly reduced fibrosis compared to *Sgcg*^*D2*^ mice (Figure 2). We also evaluated fibrosis in aged (32 week) animals and found a persistent reduction in fibrosis in the *Sgcg*^*MRL/D2*^ mice (Figure 2, diagonal bars). The 129T2/SvEmsJ strain was previously shown to suppress the severity of muscle pathology in Sgcg null mice [33]. In comparison, the MRL background suppressed fibrosis more than the 129T2/SvEmsJ background (Figure 3). The MRL background dramatically reduced fibrosis in the heart and abdominal muscles compared to 129T2/SvEmsJ. The MRL background also suppressed fibrosis more than the 129T2/SvEmsJ background in the diaphragm and quadriceps muscles, but to a lesser degree. This finding suggests that the phenotypically beneficial genetic modifiers in the MRL genome may be more potent than those in the 129T2/SvEmsJ genome.

### The MRL background does not protect against membrane leak

**Figure 4:**
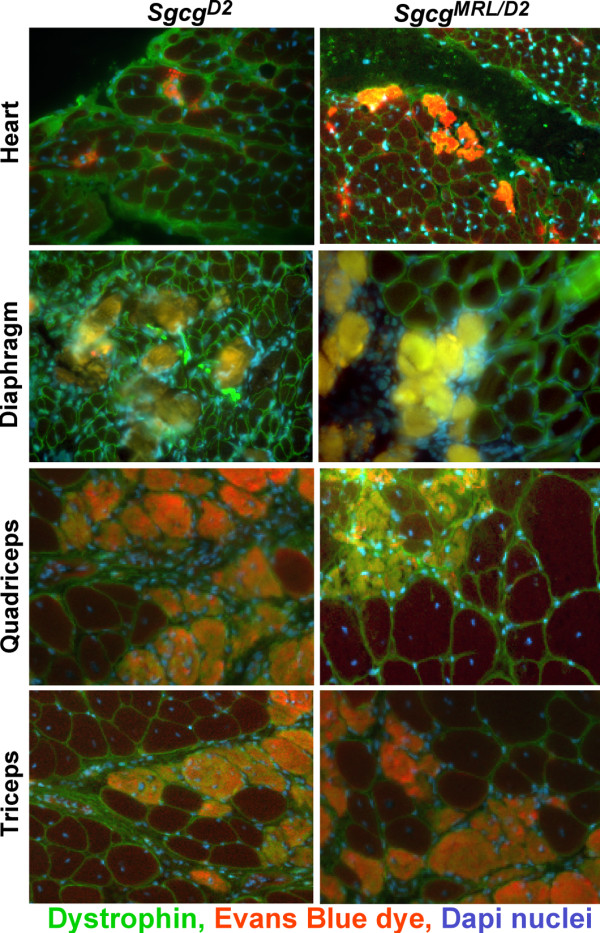
**Membrane leak is not corrected by the presence of the MRL genome in *Sgcg *heart and muscle.** Evans blue dye is found in cardiomyocytes and skeletal myofibers reflecting membrane leakiness. Dye uptake occurs in patchy pattern throughout the heart and muscle and is seen as opacified cells that fluoresce red. Nuclei are shown in blue and dystrophin in green.

**Figure 5:**
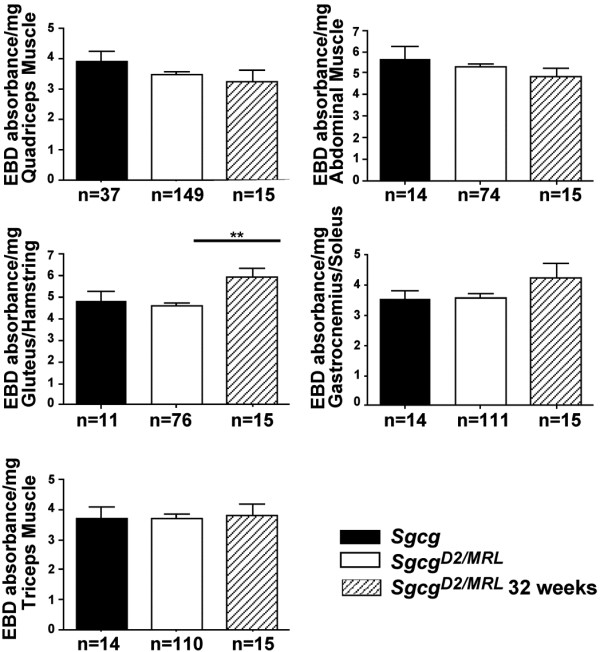
**The MRL genome does not significantly reduce membrane damage in *Sgcg *muscle.** Evans blue dye uptake is a measure of membrane damage and is represented on the y axis (absorbance/mg). In the quadriceps, triceps, abdominal and gastrocnemius/soleus muscles, membrane damage is not significantly different between *Sgcg*^D2^ animals and intercrossed *Sgcg*^MRL/D2^ animals at both the 8- and 32-week time points. In the gluteus/hamstring muscles, membrane damage is significantly (**p < 0.01) increased at the 32-week time point in intercrossed *Sgcg*^MRL/D2^ animals compared to the 8-week time point.

Disruption of the membrane-associated dystrophin complex renders the sarcolemma unusually fragile, leading to abnormal membrane leakage that is visualized by uptake of the nonspecific vital tracer Evans blue dye [20]. Upon gross inspection, the muscles from the *Sgcg*^*MRL/D2*^ mice displayed high levels of dye uptake, comparable to what was observed in the parental *Sgcg* mice (Additional file 1: Figure S1). On a microscopic level, dye-positive cells were readily detectable in *Sgcg*^*MRL/D2*^ muscle and heart (Figure 4). Evans blue dye levels were measured in multiple muscle groups and were not significantly different between *Sgcg*^*MRL/D2*^ and *Sgcg*^*D2*^ muscles for quadriceps, triceps, gastrocnemius/soleus, gluteus and abdominals muscle groups (Figure 5). We also measured dye uptake in older animals at 32 weeks to assess disease progression. At 32 weeks, the skeletal muscles continued to show comparable levels of membrane leak as what was seen at 8 weeks (Figure 5). The gluteus/hamstring group of muscles showed an increased of dye uptake compared to the 8-week animals. This increase was not seen for other muscle groups.

**Figure 6:**
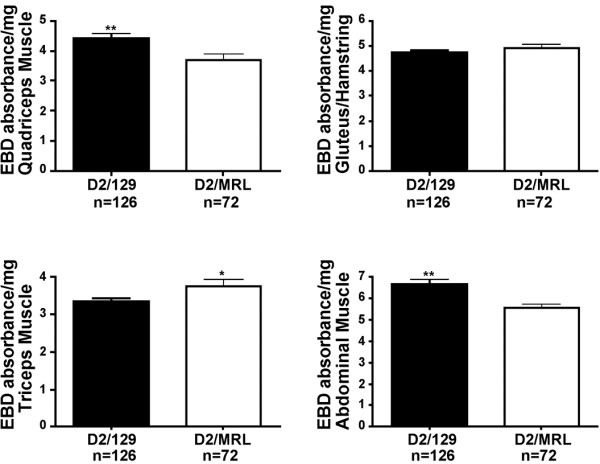
**The MRL genome has a variable ability to reduce membrane leak in muscular dystrophy compared to the 129T2/SvEmsJ strain.** Evans blue dye uptake was measured in multiple muscle groups represented on the y axis (absorbance/mg). Membrane damage is reduced in the quadriceps (**p = 0.0053) and abdominal (**p = 0.0012) muscles of intercrossed *Sgcg*^*D2/MRL*^ animals compared to intercrossed *Sgcg*^*D2/129*^ mice. Membrane damage is increased in the triceps muscle (*p = 0.0298) of intercrossed *Sgcg*^*MRL/D2*^ animals. There is no difference in membrane damage in the gluteus/hamstring muscle.

We compared suppression of dye uptake by the MRL background to that induced by the 129T2/SvEmsJ background in *Sgcg* mice (Figure 6). The quadriceps and abdominal muscles from *Sgcg* null mice with a contribution from the 129T2/SvEmsJ background showed increased membrane leak compared to *Sgcg* mice with an MRL contribution. However, the triceps muscle group from the MRL background showed increased dye uptake, and the gluteus/hamstring muscle group showed no significant difference. Thus, there was no consistent suppression of membrane leakiness by the MRL strain.

### The MRL genome may promote skeletal muscle regeneration

**Figure 7:**
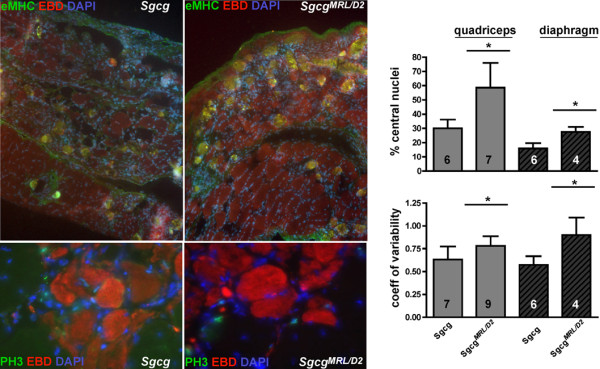
**Regeneration may be enhanced in the MRL background.** Embryonic myosin heavy chain (eMHC) staining was increased in *Sgcg*^*MRL/D2*^ compared to *Sgcg* muscle, and eMHC fibers were seen in areas where dye uptake was seen. However, phosphorylated histone 3 (PH3), a marker of mitotic index, was not increased in *Sgcg*^*MRL/D2*^ compared to *Sgcg* muscle. Skeletal muscle central nucleation and fiber size variability are enhanced by the MRL background. *Sgcg*^*MRL/D2*^ muscle demonstrated an increase in centrally nucleated fibers compared to *Sgcg*^*D2*^ mice. Fiber size variability was reflected in the coefficient of variability of fiber diameter measurements. The solid line indicates significance (*p* < 0.05). These data support that the MRL exerts its effect in skeletal muscle, at least in part, by promoting regeneration.

The MRL background is thought to exert part of its effect by promoting regeneration [34]. We examined embryonic myosin heavy chain (eMHC) expression as a reflection of regeneration. *Sgcg*^*MRL/D2*^ muscle shows patchy areas with a qualitative increase in eMHC-positive fibers compared to *Sgcg* (Figure 7), and wild-type muscle showed no eMHC positive fibers (data not shown). In dystrophic skeletal muscle, ongoing regeneration is thought to offset degeneration. Consistent with this, eMHC-positive regions were also positive for Evans blue dye uptake, indicative of muscle damage. We also evaluated phosphorylated histone 3 (PH3) as a reflection of mitotic index. No clear differences for PH3 staining were seen between *Sgcg*^*MRL/D2*^ and *Sgcg* muscle. Regenerating skeletal muscle, whether from trauma or muscular dystrophy, is identified by the presence of myofibers with centrally positioned nuclei. In muscular dystrophy, ongoing regeneration is also marked by increased fiber size variability. *Sgcg*^*MRL/D2*^ mice have an increased number of central nuclei in quadriceps and diaphragm muscle compared to *Sgcg*^*D2*^ mice (Figure 7). These same muscles also showed increased fiber size variability when there is a contribution from the MRL background. These data are consistent with a model in which favorable matrix remodeling may support enhanced regeneration.

We also assessed apoptosis using a TUNEL assay and caspase 3 staining and again found no differences between *Sgcg*^*MRL/D2*^ and *Sgcg* muscle, suggesting that gross differences in programmed cell death are unlikely to account for the improved healing of the MRL strain (Additional file 1: Figure S2). We also characterized whether T cell infiltration or macrophage infiltration differed qualitatively between *Sgcg*^*MRL/D2*^ and *Sgcg* muscle, but we found no clear differences between *Sgcg*^*MRL/D2*^ and *Sgcg* muscle, suggesting other features contribute to the MRL background’s effect on muscular dystrophy (Additional file 1: Figure S2).

### Improved cardiac function from a 50% contribution of the MRL background

We performed 2D and M mode echocardiography on *Sgcg*^*MRL/D2*^ and *Sgcg*^*D2*^ mice at 12 weeks of age. Both fractional shortening and the left ventricular ejection fraction were significantly reduced in *Sgcg*^*D2*^ mice, indicating that the increased fibrosis impairs cardiac function (Table 2). Fractional shortening and the left ventricular ejection fraction were similar between *Sgcg*^*MRL/D2*^ and *WT*^*MRL/D2*^ mice, consistent with improved function mediated by 50% of the MRL genome. Wall thickness was also increased in *Sgcg* mice compared to the wild type of the same background. In contrast, wall thickness was not different between *Sgcg*^*MRL/D2*^ and *WT*^*MRL/D2*^ mice.

**Table 2:** **Cardiac function in *Sgcg *mice**

	**Fractional shortening**
*Sgcg*^*D2*^(*n* = 4)	33.87 ± 4.80^**a**^
*WT*^*D2*^ (*n* = 5)	44.58 ± 7.65^**a**^
*Sgcg*^*MRL/D2*^ (*n* = 5)	46.00 ± 14.4
*WT*^*MRL/D2*^ (*n* = 5)	48.37 ± 11.21

^**a**^*p* = 0.046.

### Chromosome 2 associates with reduced fibrosis Sgcg heart and diaphragm muscle

**Figure 8:**
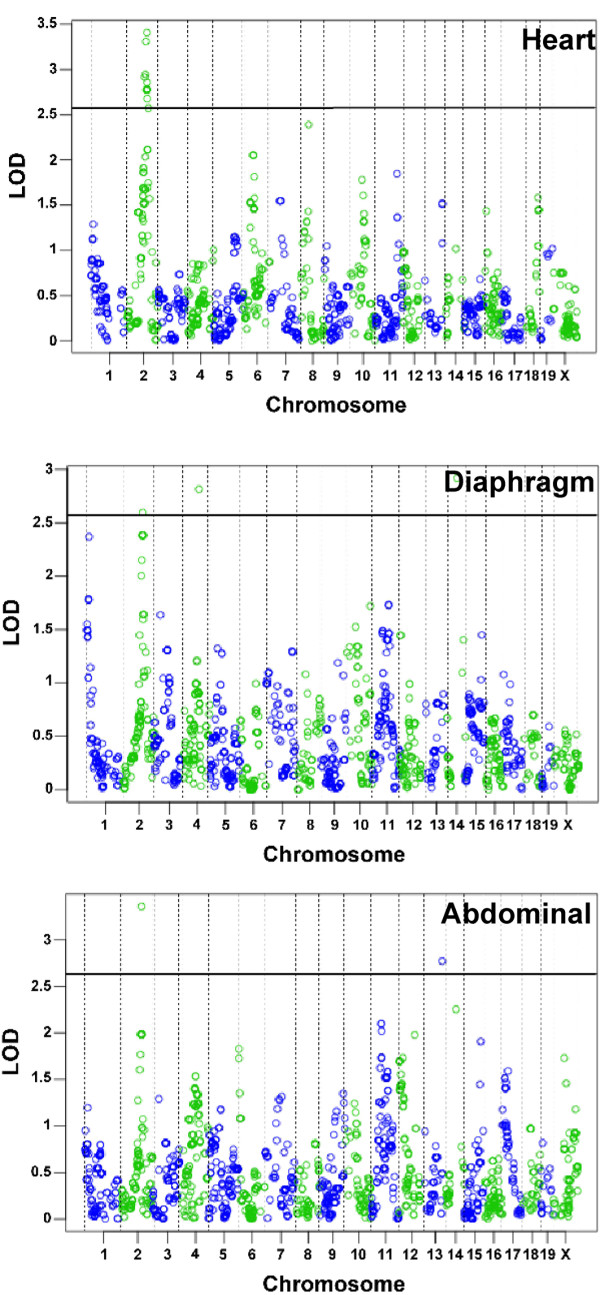
**Fibrosis in the heart, diaphragm and abdominal muscles is modified by a locus on chromosome 2.** Genome-wide association was examined for fibrosis in *Sgcg*^*MRL/D2*^ progeny in the (**A**) cardiac muscle (*n* = 65), (**B**) diaphragm (*n* = 78) and (**C**) abdominal muscle (*n* = 78). Chromosomes are plotted on the x axis, and the informative SNPs tested (*n* = 1,707) are displayed as open circles that alternate color by chromosome; LOD scores are represented on the y axis. Overlapping regions on chromosome 2 showed suggestive (*p* < 0.63) association with fibrosis in heart, diaphragm and abdominal muscle.

We conducted a genome-wide scan using markers that were informative in the two parental strains DBA/2J and MRL/MpJ (Figure 8). QTLRel was used to identify regions of association; this analysis takes into account the relatedness of individual animals in the cohort [29,30]. A region on chromosome 2 was identified that associated with fibrosis in the heart. The 1.5-LOD drop interval of this region spans from 64.8008−73.1758 cM in mouse genome build 37.1. This same region was also associated with fibrosis in the diaphragm muscle and also for fibrosis in the abdominal muscles. It should be noted that the significance of these associations is suggestive (*p* < 0.63) when using the stringent QTLRel analysis. However, the overlapping intervals found in heart, diaphragm and abdominal muscles provide additional support that this interval modifies fibrosis.

### Ltbp4 polymorphism does not account for the MRL healing properties

The DBA/2J strain contains an insertion/deletion polymorphism within the *Ltbp4* gene [21] that modifies both membrane permeability and fibrosis, as two independent traits, in *Sgcg* muscular dystrophy. *Ltbp4* encodes latent TGFβ-binding protein 4, and TGFβ proteins have been extensively linked to fibrosis in many disease states including muscular dystrophy [35,36]. Most murine strains, including the MRL/MpJ strain used here, contain the protective *Ltbp4* allele with an additional 12 amino acids inserted in exon 12. Because the *Sgcg*^*MRL/D2*^ cohort used here contained both the protective (Ltbp^I^ allele) and the disease-enhancing allele (Ltbp4^D^), we tested whether *Ltbp4* genotype correlated with fibrosis in the *Sgcg*^*MRL/D2*^ cohort. Table 3 shows that neither membrane permeability nor fibrosis is correlated with the *Ltbp4* genotype in the *Sgcg*^*MRL/DBA2J*^ cohort. Therefore, *Ltbp4* does not account for the suppressive effect of the MRL background, and other genetic modifiers account for this difference.

**Table 3:** ***Ltbp4 *genotype in *Sgcg***^***MRL/D2***^**mice**

	**Dye uptake**	**Fibrosis**
*Ltbp4*^*D/D*^ (*n* = 14)	3.90 ± 0.83	6.00 ± 1.81
*Ltbp4*^*D/I*^ (*n* = 34)	4.18 ± 0.84	8.05 ± 3.02
*Ltbp*^*I/I*^ (*n* = 47)	4.00 ± 0.85	6.64 ± 2.15

## Discussion

### The MRL genome protects against fibrosis but not membrane leak

Mutations in the dystrophin or sarcoglycan genes share a common pathological mechanism characterized by disruption of the plasma membrane of cardiomyocytes and skeletal myofibers. Membrane leak is an early step in the pathological process while fibrosis is thought to be a secondary response. Our study suggests that there are genetic pathways that target fibrosis without altering the sarcolemmal leak properties. In the heart, reduced fibrosis was correlated with functional benefit, although cardiac function may be improved from both cardiac intrinsic as well as cardiac extrinsic features. For example, improved skeletal muscle function may contribute to improved cardiac function, particularly when considering the contribution of the respiratory musculature.

The MRL strain typically shows the improved healing capability in its first 2–6 months of life. After 6 months of age, wild-type MRL mice develop autoimmune disorders; it is for this reason that we conducted studies using a 50% contribution of the MRL strain. Quantitative trait mapping has been used to define many different genetic regions associated with the healing properties [14]. The autoimmune properties have been genetically separated from the healing properties since the four to six different “autoimmune” genetic loci do not overlap with those associated with improved healing. In our studies, only 50% of the MRL genome was capable of suppressing fibrosis and improving function. In the course of these studies, we never identified a single *Sgcg*^*MRL/D2*^ animal that had markedly elevated fibrosis. This stands in contrast to what is normally observed in the *Sgcg* animals where outliers with markedly elevated fibrosis are often seen. The maximum values for fibrosis in *Sgcg*^*MRL/D2*^ mice were all well below what was measured in *Sgcg*^*D2*^ mice. This is consistent with multiple, dominant genetic loci imparting the improved healing capabilities, similar to what has been observed for the ear hole repair properties in the MRL strain [37]. The finding that improvement persists in the *Sgcg*^*MRL/D2*^ mice, with almost complete suppression of fibrosis at 32 weeks, suggests that a persistent healing effect can arise from the MRL strain.

### Chromosome 2 candidate genes

When considering the overlapping region on chromosome 2 from the heart, diaphragm and abdominal fibrosis data, the interval contains 49 known genes. There are additional predicted and unnamed genes and genes encoding olfactory receptors that are not considered likely candidates. Within the interval is *Jag1*, encoding jagged the ligand for the Notch receptor. Loss of function of Notch 3 leads to muscle hyperplasia, especially when subjected to repetitive injury [38]. Ex vivo activation of Notch signaling helps maintain donor cell engraftment during myoblast transfer [39]. Another gene linked to growth and healing in the interval is *Bmp2*-encoding bone morphogenetic protein 2. The BMPs mediate musculoskeletal regeneration [40], and given the MRL background effect on multiple cells and tissues, BMP2 is well positioned to contribute to the MRL superhealing response. That said, this chromosome 2 interval has not previously been linked to other MRL healing properties. The strict criteria imposed by the QTLRel algorithm makes it unlikely that these results derive from relatedness of animals within the cohort.

### Cardiac injury and repair in the MRL strain

The ability of the heart to recover after injury has been studied in the MRL strain using cryoinjury, left anterior descending ligation and ischemia reperfusion methods [5,6,8-10,14,15]. However, scar reduction has been noted after some forms of injury while not after others. In common to all these studies is an acute injury model where a normal heart was substantially damaged in a single setting. The MRL’s ability to heal the heart may be limited such that larger amounts of injury may be insurmountable, as proposed by Naseem [6]. Our data support that lower, although persistent, levels of injury can be managed by the MRL strain where there is significant reduction in fibrosis and a corresponding functional improvement in cardiac function.

### Mechanisms for MRL healing

A number of mechanisms likely act in concert to achieve suppression of fibrosis and improvement of function. In skeletal muscle, where muscle stem cells robustly regenerate muscle after injury, there is indirect evidence of enhanced regeneration. Specifically, there is an increase in centrally nucleated myofibers, which is thought to reflect enhanced myoblast fusion. However, our data do not distinguish whether the MRL background exerts its effect on muscle regenerative cells, or by creating a more supportive extracellular matrix or both. MRL animals heal with embryonic characteristics with enhanced blastema formation and metabolic and gene expression features consistent with an earlier developmental state [41]. Altered protease expression has also been noted in the MRL mouse in response to damage [5]. Recent work characterizing the immune infiltrate in muscular dystrophy found that reducing osteopontin was effective at suppressing fibrosis [17]. Together these data may favor matrix-associated modifications that can modulate muscle fibrosis and function.

## Conclusions

Herein the MRL background was shown to reduce fibrosis in a chronic model of muscular dystrophy and cardiomyopathy, the *Sgcg* mouse. Although membrane leak was still evident in *Sgcg* mice sharing a portion of the MRL genome, the reduction in fibrosis was associated with improved cardiac function. The identification of gene(s) from the MRL genome will help identify pathways important for chronic repair of myopathic processes.

## Abbreviations

BMP: Bone morphogenetic protein; D2: (DBA/2J); DMD: Duchenne muscular dystrophy; HOP: Hydroxyproline; LTBP4: Latent TGFβ-binding protein; MRL: Murphy Roth Large; QTL: Quantitative trait loci; SNP: Single nucleotide polymorphism; TGFβ: Transforming growth factor β.

## Competing interest

The authors have no competing interests related to this work.

## Authors’ contributions

AH conducted the phenotypic analysis of muscle. KAS conducted the genome-wide SNP analysis and phenotype analysis. GK performed the echocardiographic analysis. MH oversaw the breeding. JHC assisted with phenotypic analysis. EMM conceived the experiments, analyzed the data and wrote the manuscript. All authors read and approved the final manuscript.

## Supplementary Material

Additional file 1 **Figure S1.** Shown are gross images from *Sgcg* vs. *Sgcg*^*MRL/D2*^ mice. Evans blue dye uptake could be readily seen in the quadriceps and diaphragm muscles and did not appear grossly altered by the presence of the MRL background. In contrast, fibrosis was visually reduced in the quadriceps and heart of *Sgcg*^*MRL/D2*^ compared to *Sgcg* mice. The diaphragm muscle retained evidence of fibrosis in *Sgcg*^*MRL/D2*^, but the white stripes of fibrosis were smaller, and intact diaphragm muscle was still evident compared to the near total replacement of diaphragm muscle in *Sgcg* mice. **Figure S2.** Shown is staining for apoptosis with TUNEL and caspase indicating no gross differences between *Sgcg* and *Sgcg*^*MRL/D2*^ muscle. CD3 and MAC1 staining to examine T cell and macrophage infiltrate also did not appear grossly altered by the presence of the MRL background. Click here for file
