# Supplementary material for: The superhealing MRL background improves muscular dystrophy
Source: Skelet Muscle. 2012 Dec 5;2:26. doi: 10.1186/2044-5040-2-26 (PMC3534636; doi:10.1186/2044-5040-2-26)
Supplement: Additional file 1 — Figure S1. Shown are gross images from Sgcg vs. SgcgMRL/D2 mice. Evans blue dye uptake could be readily seen in the quadriceps and diaphragm muscles and did not appear grossly altered by the presence of the MRL background. In contrast, fibrosis was visually reduced in the quadriceps and heart of SgcgMRL/D2 compared to Sgcg mice. The diaphragm muscle retained evidence of fibrosis in SgcgMRL/D2, but the white stripes of fibrosis were smaller, and intact diaphragm muscle was still evident compared to the near total replacement of diaphragm muscle in Sgcg mice. Figure S2. Shown is staining for apoptosis with TUNEL and caspase indicating no gross differences between Sgcg and SgcgMRL/D2 muscle. CD3 and MAC1 staining to examine T cell and macrophage infiltrate also did not appear grossly altered by the presence of the MRL background. [file 2044-5040-2-26-S1.pdf]

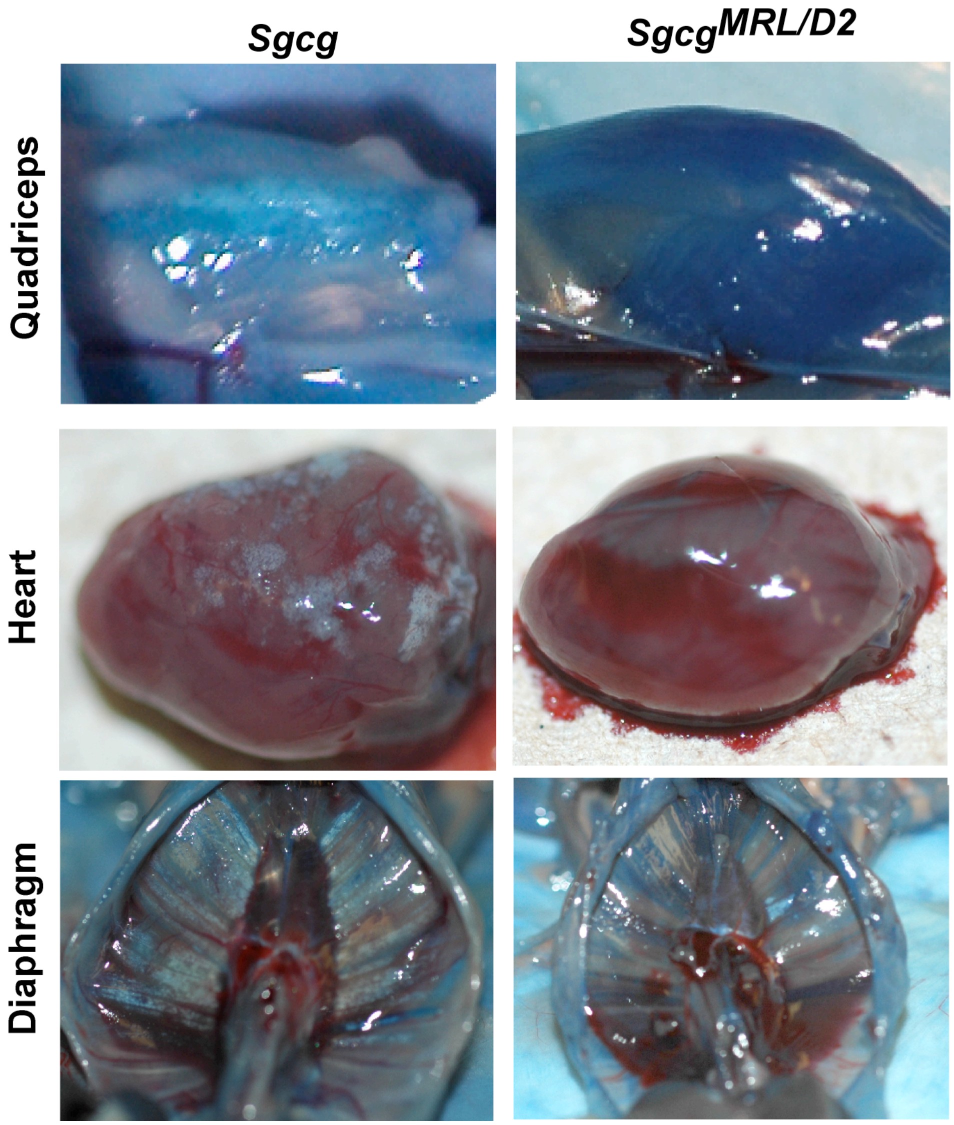

Supplementary Figure 1. Shown are gross images from *Sgcg* vs. *Sgcg*$^{MRL/D2}$ mice. Evans blue dye uptake could be readily seen in the quadriceps and diaphragm muscles and did not appear grossly altered by the presence of the MRL background. In contrast, fibrosis was visually reduced in quadriceps and the heart of *Sgcg*$^{MRL/D2}$ compared to *Sgcg* mice. The diaphragm muscle retained evidence of fibrosis in *Sgcg*$^{MRL/D2}$, but the white stripes of fibrosis were smaller and intact diaphragm muscle was still evident compared to the near total replacement of diaphragm muscle in *Sgcg* mice.

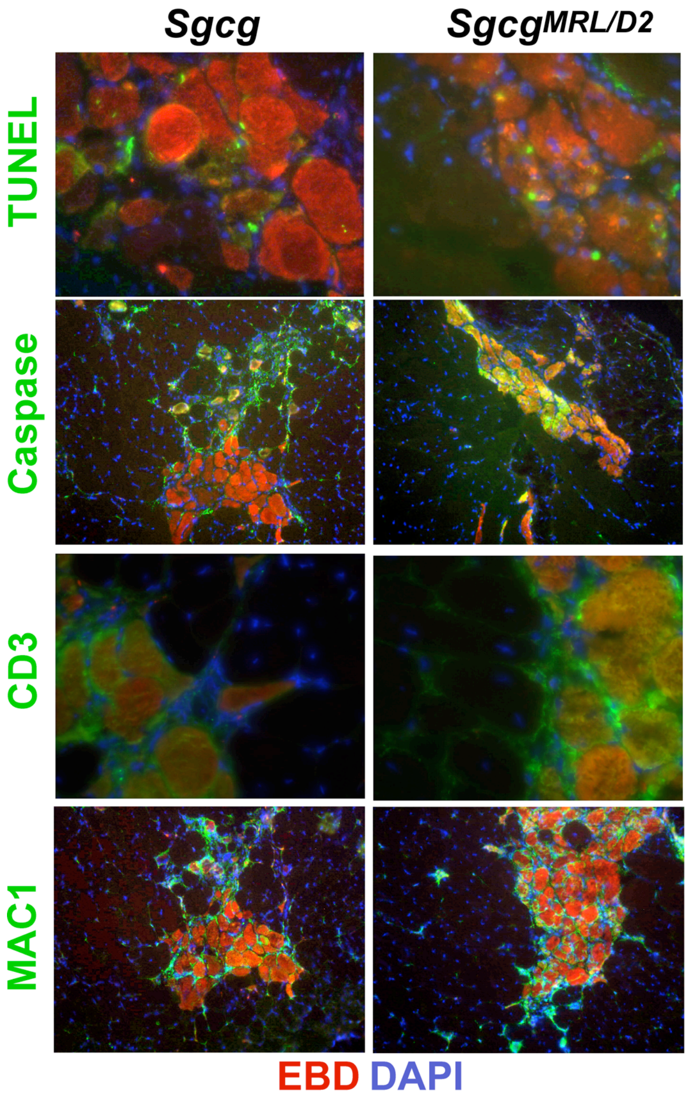

Supplementary Figure 2. Shown is staining for apoptosis with TUNEL and caspase indicating no gross differences between *Sgcg* and *Sgcg*$^{MRL/D2}$ muscle. CD3 and MAC1 staining to examine T cell and macrophage infiltrate also did not appear grossly altered by the presence of the MRL background.
